# A unique case of two somatic *APC* mutations in an early onset cribriform-morular variant of papillary thyroid carcinoma and overview of the literature

**DOI:** 10.1007/s10689-019-00146-4

**Published:** 2019-10-09

**Authors:** M. D. Aydemirli, K. van der Tuin, F. J. Hes, A. M. W. van den Ouweland, T. van Wezel, E. Kapiteijn, H. Morreau

**Affiliations:** 1 Department of Medical Oncology, Leiden University Medical Center, P.O. Box 9600, 2300 RC Leiden, The Netherlands; 2 Department of Clinical Genetics, Leiden University Medical Center, Leiden, The Netherlands; 3 Department of Clinical Genetics, Erasmus University, Rotterdam, The Netherlands; 4 Department of Pathology, Leiden University Medical Center, Leiden, The Netherlands

**Keywords:** Cribriform-morular, Thyroid carcinoma, Cribriform-morular variant papillary thyroid carcinoma, *APC*, β-catenin, Wnt, Familial adenomatous polyposis, FAP

## Abstract

We report a case of a 22-year-old female patient who was diagnosed with a cribriform-morular variant of papillary thyroid carcinoma (CMV-PTC). While at early ages this thyroid cancer variant is highly suggestive for familial adenomatous polyposis (FAP), there was no family history of FAP. In the tumor biallelic, inactivating *APC* variants were identified. The patient tested negative for germline variants based on analysis of genomic DNA from peripheral blood leukocytes. Somatic mosaicism was excluded by subsequent deep sequencing of leukocyte and normal thyroid DNA using next generation sequencing (NGS). This report presents a rare sporadic case of CMV-PTC, and to the best of our knowledge the first featuring two somatic *APC* mutations underlying the disease, with an overview of CMV-PTC cases with detected *APC* and *CTNNB1* pathogenic variants from the literature.

**Electronic supplementary material:**

The online version of this article (10.1007/s10689-019-00146-4) contains supplementary material, which is available to authorized users.

## Introduction

The cribriform-morular variant of papillary thyroid carcinoma (CMV-PTC) is a rare subtype of differentiated thyroid cancer and generally has a good prognosis [1]. CMV-PTC is highly associated with heterozygous germline *APC* mutations leading to familial adenomatous polyposis (FAP) [2, 3]. FAP, an autosomal dominant disorder, is characterized by multiple adenomatous colorectal polyps, often showing progression into adenocarcinoma and predisposition for a large spectrum of extracolonic tumors, including thyroid cancer. De novo *APC* mutations are reported in 11–25% of FAP patients [4, 5]. About 39–53% of reported CMV-PTC cases in literature were found to harbor a germline *APC* variant or were clinically diagnosed with FAP [6, 7]. However, CMV-PTC may also occur sporadically in the absence of FAP.

CMV-PTC has a distinctive histologic morphology featuring morules and a cribriform growth pattern, which is related to the permanent activation of the Wnt pathway, and reflected by nuclear β-catenin staining on immunohistochemistry (IHC) [1, 8]. The latter may result from biallelic *APC* gene inactivation, or from somatic mutations of the *β-catenin* (*CTNNB1*) [8–12] or *AXIN1* gene (or combinations of gene variants), that are functionally similar [1, 13]. As the *APC* gene acts as a negative regulator of the Wnt pathway, mutated *APC* may result in a truncated protein lacking the majority of β-catenin binding sites, consequentially being unable to degrade β-catenin along with cytoplasmic and nuclear storage, while regulation of the latter is critical to the tumor suppressive effect of *APC* [14].

Here we present an extremely rare case of a young woman with sporadic CMV-PTC, in whom biallelic somatic inactivating *APC* variants were detected.

## Case description

A 22-year-old female with an unremarkable medical history and negative family history for thyroid disease, presented with a palpable thyroid mass. Ultrasonography revealed a solitary thyroid nodule, measuring 1.5 cm by 1.8 cm by 2.1 cm, located on the right lobe, with an isoechoic and hyper-vascular composition. Cytologic findings on fine-needle aspiration (FNA) of the nodule were suggestive of PTC (Bethesda V). Total nucleic acid (undivided DNA and RNA) was isolated from FNA material using a fully automated extraction procedure [15]. No somatic DNA variants were identified upon analysis with a customized NGS AmpliSeq Cancer Hotspot Panel which includes well known thyroid carcinoma driver genes (e.g. *BRAF*, *NRAS*, *HRAS*, *KRAS*, *TP53*, *PIK3CA* and *CTNNB1*). A total thyroidectomy was performed with intraoperative frozen-section biopsy that was concordant with FNA findings. Histologically, the encapsulated tumor was highly cellular and composed of a combination of trabecular, solid, cribriform and follicular growth patterns with morules (Online Resource 1). Immunohistochemical (IHC) analysis for β-catenin, performed as previously described [16], showed both positively stained nuclei and cytoplasm, indicative of activation and characteristic for CMV-PTC [3].

*APC* was sequenced as previously described [17], on tumor DNA extracted from formalin-fixed, paraffin-embedded (FFPE) tissue cores.

Biallelic, class 4 (likely pathogenic) and class 5 (pathogenic), respectively, somatic inactivating *APC* variants were identified (NM_000038.5): c.3124delA, p. (Ser1042Valfs*14) and c.3183_3187delACAAA, p. (Gln1062*) (Online Resource 2).

To explore the chances for having FAP based on these findings, the patient was referred for genetic counselling. There was no family history of any FAP related tumors, in particular, no colon cancer or colonic polyposis. Genomic DNA was extracted from peripheral blood leukocytes according to standard procedures using Sanger sequencing and multiplex ligation-probe amplification (MLPA). All 15 exons of the *APC* gene tested negative for germline variants. Subsequent screening of DNA from leukocytes and normal thyroid tissue for the two somatic *APC* variants was performed using NGS deep sequencing (coverage of the variant region minimally 1000 ×). The specific variants were not identified in the leukocyte DNA or normal thyroid, excluding somatic mosaicism. Therefore, referral for endoscopic surveillance, as well as genetic counselling of related family members was considered unwarranted. As standard of care, the patient received complementary ablation therapy with radioactive iodine. The patient had a total remission and also no recurrence was noted during follow up.

## Literature overview

In Table 1 an overview of pathogenic variants in *APC* or *CTNNB1* genes detected in 44 cases of CMV-PTC patients reported in literature is listed (Table 1). We conducted a Pubmed search on English literature using a combination of the terms: cribriform-morular, cribriform or morul* combined with thyroid carcinoma. Most of selected papers were reported in the reviews by Lam et al. [7] and/or Pradhan et al. [6] and additional relevant papers were found through cross-referencing. Reported variants in literature linked to the Catalogue of Somatic Mutations in Cancer (COSMIC) database (https://cancer.sanger.ac.uk/cosmic) and NCBI ClinVar (https://www.ncbi.nlm.nih.gov/clinvar/) or variants that could be retrieved from Leiden Open-source Variation Database (LOVD) (http://www.lovd.nl/3.0/home) were listed and annotated according to the Human Genome Variation Society (HGVS) guidelines for nomenclature (http://www.hgvs.org/content/guidelines).

**Table 1:** Overview of likely pathogenic *APC* and *CTNNB1* gene variants in CMV-PTC patient cases reported in literature

Sex age	Germline pathogenic *APC* variant	Exon	T	Somatic pathogenic *APC* variant	Exon	LOH	Somatic pathogenic *CTNNB1* variant	Exon	References
F 23 yr	–		T1	–		–	c.65T>C, p.(Val22Ala)	3	[9]
			T2	–		–	c.166G>A, p.(Asp56Asn)	3	
F 20 yr	–			–		ND	c.110C>T, p.(Ser37Phe)	3	[8]
F 34 yr	–			–		–	c.85T>C, p.(Ser29Pro)	3	[9]
F 22 yr	–			–		–	c.160G>A, p.(Glu54Lys)	3	[9]
F 23 yr	ND			ND		ND	c.115G>A, p.(Ala39Thr)	3	[9]
F 30 yr	c.1538delT, p.(Val513Glufs*10)	11	T1	–		–	c.145A>G, p.(Lys49Glu)	3	[9, 18]
			T2	–		–	c.131C>T, p.(Pro44Leu)	3	
F 29 yr	Whole gene deletion			c.1548+1G>A, splice site variant^d^	e	+	ND		[19]
F 25 yr	c.1660C>T p.(Arg554*)	13	T1	c.922delC, p.(Leu308fs*28)	8	–	–		[20]
			T2	c.2706_2725del20, p.(Glu902fs*3)	15	–	–		
			T3	c.1821delT, p.(Cys607fs*3)	14	–	–		
			T4	c.1920delG, p.(Asn641fs*5)	14	–	–		
			T5	c.2803_2804insA, p.(Tyr935fs*1)	15	–	–		
			T6	c.1602delA, p.(Lys534fs*15)	12	–	–		
F 20 yr	c.3329C>G, p.(Ser1110*)	15	T1	c.3180_3184delAAAAC, p.(Gln1062fs*1)	15	ND	ND		[21, 22]
			T2	c.2569G>T, p.(Gly857*)	15	ND	ND		
F 26 yr	c.524delC, p.(Thy175Metfs*10)	4	T1	c.2656C>T, p.(Gln886*)	15	–	ND		[21, 22]
			T2	c.4606G>T, p.(Glu1536*)	15	–	ND		
			T3	c.4666_4667insA, p.(Thr1556fs*3)	15	–	ND		
			T4			+	ND		
			T5			+	ND		
F 24 yr	c.2093T>G, p.(Leu698*)	15		c.4362_4567ins159, p.(Lys1454fs*3)	15	ND	ND		[11]
F 21 yr^a^	c.832C>T, p.(Gln278*)	7		c.1363_1378delinsTTTCTC, p.(Lys455Phefs*9)	10	ND	ND		[23]
F 48 yr^a^	c.832C>T, p.(Gln278*)	7		–		ND	ND		[23]
M 42 yr	Duplication	2/3		–		ND	–		[12]
F 27 yr	c.1917insA, p.(Arg640Thrfs*11)	14		ND		ND	ND		[24]
F 40 yr	c.3149delC, p.(Ala1050Glufs*6)	15		ND		ND	ND		[24]
F 32 yr	‘abnormal splicing in exon 9’; molecular defect not identified	9		ND		ND	ND		[18, 25]
F 29 yr	c.3927_3931del, p.(Glu1309Aspfs*4)	15		ND		ND	ND		[26]
F 30 yr	c.419_422del, p.(Glu140Glyfs*28)	3		–		ND	ND		[27]
F 19 yr	c.1775T>G p.(Leu592*)	14		–		ND	ND		[27]
F 22 yr	c.2336del p.(Leu779*)	15		–		ND	ND		[27]
F 18 yr	c.2928_2929del, p.(Gly977Serfs*7)	15		–		ND	ND		[27]
F 27 yr	c.2979del, p.(Lys993Asnfs*12)	15		–		ND	ND		[27]
F 39 yr	c.3183_3187del, p.(Gln1062*)	15		–		ND	ND		[27]
F 26 yr	c.3927_3931del, p.(Glu1309Aspfs*4)	15		–		ND	ND		[27]
F 22 yr^b^	c.3183_3187del, p.(Gln1062*)	15		–		ND	ND		[27]
F 20 yr^b^	c.3183_3187del, p.(Gln1062*)	15		–		ND	ND		[27]
F 36 yr^b^	c.3183_3187del, p.(Gln1062*)	15		–		ND	ND		[27]
F 24 yr	c.3183_3187del, p.(Gln1062*)	15		–		ND	ND		[27]
F 20 yr	c.3927_3931del, p.(Glu1309Aspfs*4)	15		–		ND	ND		[27]
F 27 yr	c.3927_3931del, p.(Glu1309Aspfs*4)	15		–		ND	ND		[27]
F 20 yr	c.3183_3187del, p.(Gln1062*)	15		ND		ND	ND		[28]
F 38 yr	c.2093T>A, p.(Leu698*)	15		–		ND	ND		[11]
F 49 yr	c.937_938delGA, p.(Glu313Asnfs*)	9		–		ND	ND		[11]
F 16 yr^c^	c.254A>T, p.(Lys848*)	15		ND		ND	ND		[29, 30]
F 12 yr^c^	c.254A>T, p.(Lys848*)	15		ND		ND	ND		[29, 30]
F 18 yr	c.3183_3187del, p.(Gln1062*)	15		ND		ND	ND		[31]
F 30 yr	c.3317delG, p.(Gly1106Glufs*20)	15		ND		ND	ND		[32]
F	c.2211C>G, p.(Tyr737*)	15				ND	ND		[33]
F 40 yr	Unknown variant in codon 1219	15		–		ND	ND		[27]
F 19 yr	Unknown variant in codon 1219	15		–		ND	ND		[27]
F 35 yr	–			c.1559_1563delGCTCT, p.(Cys520fs*15)	12	ND	–		[34]
F 19 yr	–			c.3927_3931delAAAGA, p.(Glu1309fs*4)	15	ND	ND		[35]
F 27 yr	–			c.3927_3931delAAAGA, p.(Glu1309fs*4)	15	ND	ND		[36]

References are listed in the appendix. Data in the table are ordered according to somatic *CTNNB1* mutations, then the germline *APC* variants (either coinciding with somatic mutations or without other mutations) and somatic *APC* mutations reported in literature. Within the list, a reverse chronological order has been pursued with annotation of the variants according to HGVS guidelines. The majority of somatic variants were found in the COSMIC database. Printed underlined: Germline variants found in ClinVar. The remaining variants were found in LOVD. Variants reported were curated and annotated using the APC reference sequence NM_000038.5

– No variants detected, *bp* base pair, *del* deletion, *F* female, *M* male, *ND* not determined, *T1*, *T2*, etc. number of tumor foci, *yr* years old

^a^Related cases (mother, daughter) belonging to the same kindred

^b,c^Related cases (sisters) belonging to the same kindred, respectively

^d^Somatic variants not found in COSMIC database

^e^1 base pair downstream of exon 12

Pathogenic *APC* variants were described in 39 cases. Of these, 36 cases had a germline *APC* variant including one whole gene deletion. Six of those cases with a germline *APC* variant were shown to harbor one additional somatic *APC* variant or per tumor nodule a distinct variant or LOH. One case with a germline *APC* variant harbored two concurrent somatic *CTNNB1* variants at a different tumor site each. Three cases were reported with one somatic *APC* mutation solely. *APC* germline variants were located between codons 140 and 1309 and *APC* somatic variants between codons 308–1556. Only a limited number of cases were analyzed for LOH of the *APC* gene [9, 12, 20–22].

Six cases have been reported with somatic *CTNNB1* mutations, comprising 8 different variants of *CTNNB1*, all of them located on exon 3. Four cases harbored single somatic *CTNNB1* variants. One case harbored two somatic *CTNNB1* mutations, both in different tumor nodules.

Reported mutations occurring in other than the aforementioned genes in Table 1, include two somatic mutations in exon 7 and 1 of *AXIN1*, that codes for a scaffold protein in the multimolecular complex that is formed by the APC protein with β-catenin and glycogen synthase kinase 3 β (GSK-3β), in a familial and a sporadic case of CMV-PTC, respectively [1, 13]. Furthermore, one apparently sporadic 45-year-old female patient case with CMV-PTC and a somatic *TERT* promoter mutation (c. 124C>T) showed an aggressive disease course, in absence of an *APC* mutation; *CTNNB1* was not evaluated in this case [37]. *RET*/PTC rearrangements have also been reported in sporadic CMV-PTC [38], and in FAP associated cases [11, 12]. High rates of *RET*/PTC gene activation have been reported by Cetta et al. [39] in cases with heterozygous *APC* genes, although somatic mutations were not determined [22], with hypotheses of a tissue-specific dominant effect [40]. Somatic *PIK3CA* c.1634 A>C (p.E545A) mutations were reported in three sporadic CMV-PTC cases of female patients aged 14, 16, 17 years [41], and suggested as a potential candidate gene involved in sporadic CMV-PTC tumorigenesis in absence of a *CTNNB1* mutation, however, *APC* gene mutation data are lacking. A 16-year old female FAP patient was reported with a somatic *KRAS* mutation (c. 181C>A (p. Q61K)); however, data on *APC* (or *CTNNB1*) genes were not reported [42].

## Discussion

In the present report we describe a young adult patient with cribriform-morular variant of PTC with biallelic somatic inactivating *APC* variants. To the best of our knowledge, it represents the first case of two pathogenic somatic *APC* variants explaining the disease occurrence.

The class 5 *APC* variant c.3183_3187delACAAA, p. (Gln1062*), has previously been described as a germline pathogenic variant in a FAP patient with PTC [43]. The other *APC* variant c.3124delA, p. (Ser1042Valfs*14) was not reported before, but was considered a class 4 (likely pathogenic) variant. The pathogenicity of variants is annotated in classes 1 to 5, with a class 4 variant being likely pathogenic and a class 5 variant being (well-known) pathogenic [44], based on literature (Pubmed) search and common or locus specific databases (Mycancergenome, Alamut Visual, NCBI dbSNP, NCBI ClinVar, COSMIC, Jackson laboratory database, LOVD, MD Anderson database).

Also, the finding of a solitary nodule in our patient, is in line with its usual appearance in sporadic cases [1].

The detection of the biallelic inactivating mutations is in line with the Knudson “two-hit hypothesis” [45], supporting the underlying nature for the tumor.

Germline variants in *APC* are frequently found in FAP patients, but absent in the *CTNNB1* gene [46, 47]. The occurrence of a germline *CTNNB1* variant has only been reported as an inactivating mutation, constituting another distinct phenotype without tumor manifestations, in two siblings, of whom the parents most likely harbored germline mosaicism [48]. Cetta et al. reported that biallelic inactivation of *APC* is usually lacking in thyroid carcinoma cases occurring in FAP [49]. The latter might be suggestive of a conveyance of a general susceptibility to thyroid tumorigenesis [50]. On the other hand, this could also be partly due to a limited or a lack of mutational analysis of the *APC* and/or *CTNNB1* gene (indicated as ND, not determined, in Table 1).

The *CTNNB1* variants in the cases listed in the overview (Table 1) were all located on exon 3, which is typically associated with β-catenin translocation from membrane to nucleus and Wnt pathway activation [51].

The majority of the reported somatic and germline *APC* variants in CMV-PTC (Table 1, [27]), were not within the mutation cluster region (MCR) in *APC* (codons 1286–1513) for somatic mutations in colorectal tumors [52]. Of the reported 17 somatic *APC* variants, 3 variants occurred in, 12 before and 2 after the MCR, respectively (Table 1). Of the reported 36 germline *APC* variants, 4 occurred in and 31 before the MCR (one of the germline variants was a whole gene deletion) (Table 1).

However, all germline *APC* variants (Table 1) were within the region extending from codons 140 to 1309, that has been associated to PTC in terms of genotype-phenotype correlations of extra-intestinal manifestations of FAP [39, 53, 54]. Of the 17 reported somatic *APC* variants (Table 1), 3 variants were out of and 14 variants were in this region (codons 140–1309), as well as the two somatic *APC* variants identified in the index patient.

In conclusion, in the current study, we report biallelic somatic (rather than germline) pathogenic *APC* variants in a young female CMV-PTC patient. Our report corroborates current ideas regarding the molecular background in CMV-PTC tumors. The true somatic nature of the variants found, was rendered most likely, using deep *APC* sequencing of leukocyte and normal DNA to exclude mosaicism. Accordingly, endoscopy was not performed. With a substantial share of FAP patients having a de novo APC mutation [4, 5], the presently reported approach conveys added value and clinical relevance especially in patients with an absent family history of FAP. As much so in patients without any evidence of detected FAP as of yet, with about 60% of total CMV-PTC being FAP associated, of whom a substantial proportion is preceded by that of thyroid cancer [1].

## Electronic supplementary material

Below is the link to the electronic supplementary material.

Supplementary material 1 (DOCX 5228 kb)
