# Supplementary material for: A unique case of two somatic APC mutations in an early onset cribriform-morular variant of papillary thyroid carcinoma and overview of the literature
Source: Fam Cancer. 2019 Oct 9;19(1):15–21. doi: 10.1007/s10689-019-00146-4 (PMC7026211; doi:10.1007/s10689-019-00146-4)
Supplement: Supplementary file 1 — Supplementary material 1 (DOCX 5228 kb) [file 10689_2019_146_MOESM1_ESM.docx]

**Electronic supplementary material**

*Familial cancer*

A unique case of two somatic *APC* mutations in an early onset cribriform-morular variant of papillary thyroid carcinoma and overview of the literature

Aydemirli MD^1^, van der Tuin K^2^, Hes FJ^2^, van den Ouweland AMW^3^, van Wezel T^4^, E. Kapiteijn^1^, Morreau H^4^.

*E-mail address of corresponding author:* M.D.Aydemirli@lumc.nl


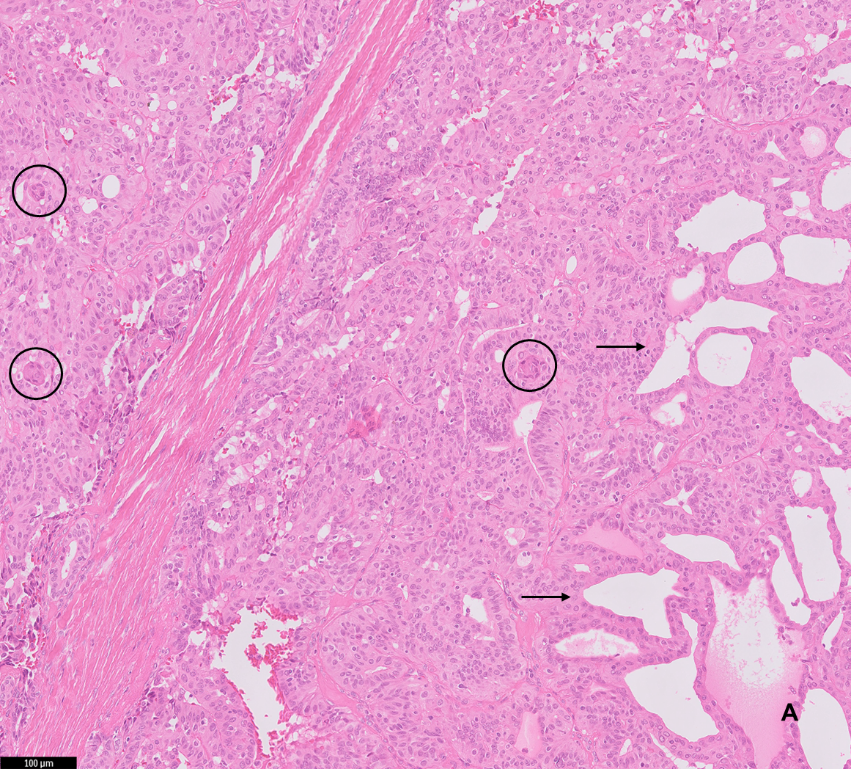


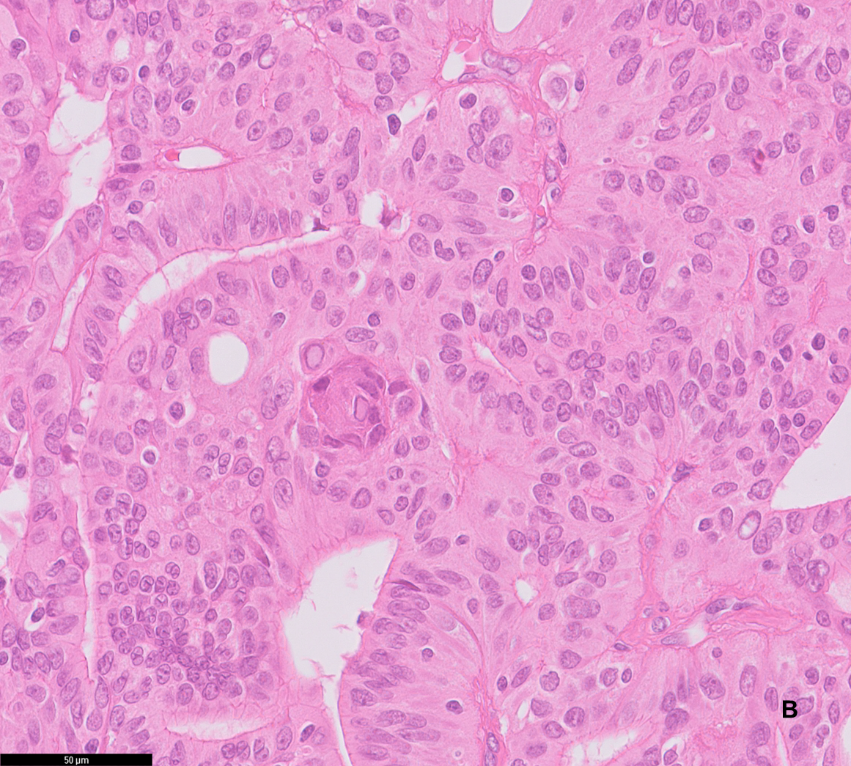


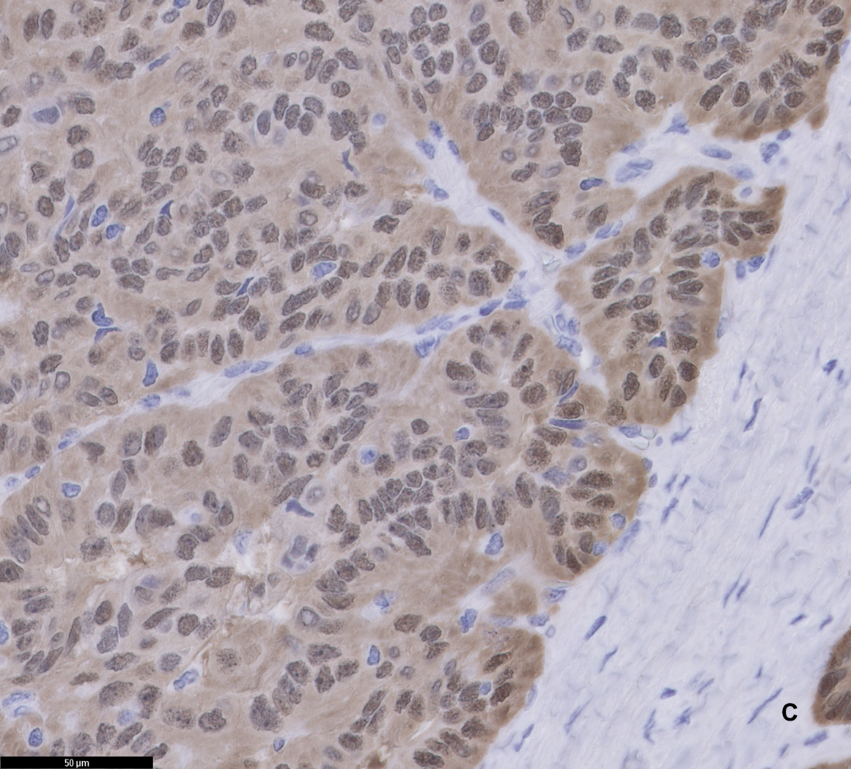


**Online Resource 1** Histologic photomicrographs of cribriform-morular variant of papillary thyroid carcinoma, (**A, H&E**) detailed characteristics of cribriform growth pattern (indicated by arrows), and morules (scattered squamoid islands indicated by circles) and (**B, H&E**) a higher magnification view of a morula with nuclear clearing (Orphan Annie-eye nuclei). (**C**) Positive immunohistochemical staining for β-catenin (1:250, #610154, BD Transduction Laboratories, Bedford, MA, USA), a hallmark feature of CMV-PTC (endothelial cells are negative; internal control).


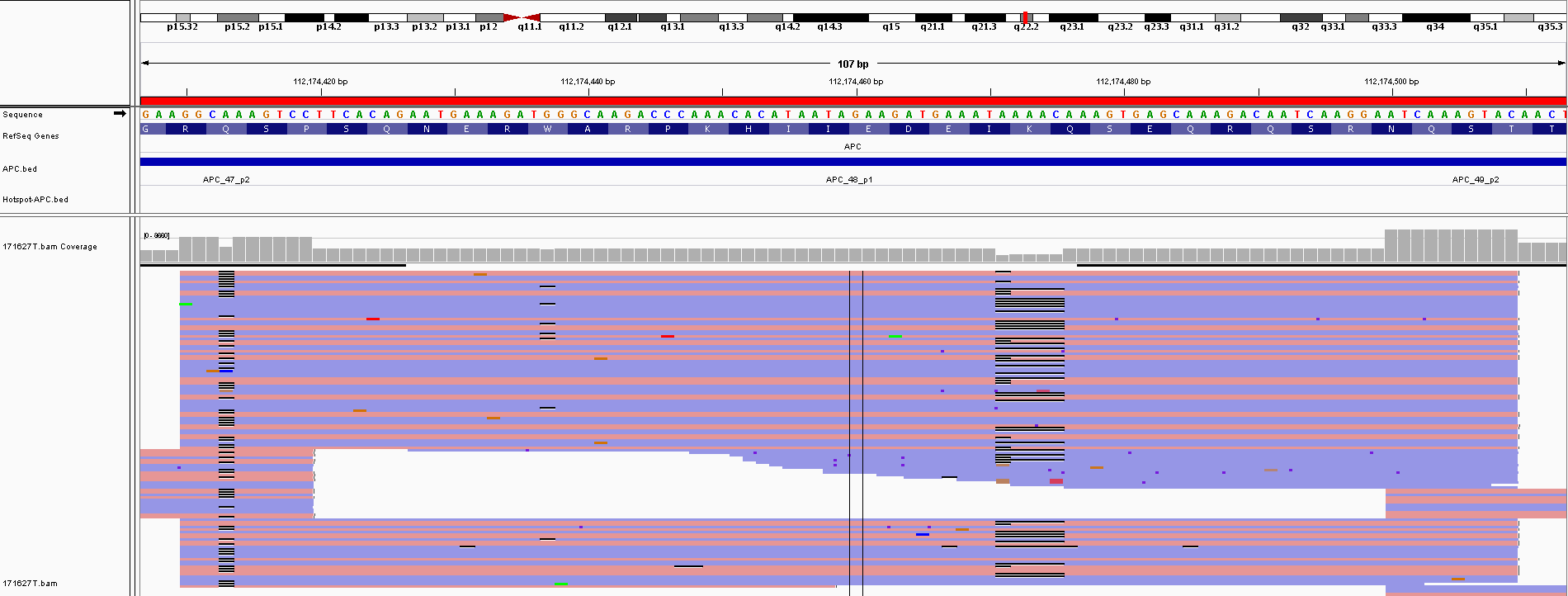


**Online Resource 2** Reads depicting the biallelic *APC* variants detected in the index patient’s tumor DNA. The *APC* variant c.3124delA, p. (Ser1042Valfs*14) is visualized on the left and the *APC* variant c.3183_3187delACAAA, p. (Gln1062*) is visualized on the right, using Integrative Genomics Viewer (IGV) (Broad Institute, MA, USA). The two variants occur within the same amplicon, on two different alleles.
